# *In Vitro* Antiophidian Properties of *Dipteryx alata* Vogel Bark Extracts

**DOI:** 10.3390/molecules15095956

**Published:** 2010-08-30

**Authors:** Virgínia Sbrugnera Nazato, Leandro Rubem-Mauro, Nathalia Aparecida Gatto Vieira, Dimas dos Santos Rocha-Junior, Magali Glauzer Silva, Patricia Santos Lopes, Cháriston André Dal-Belo, José Carlos Cogo, Marcio Galdino dos Santos, Maria Alice da Cruz-Höfling, Yoko Oshima-Franco

**Affiliations:** 1 Universidade de Sorocaba, UNISO, Rodovia Raposo Tavares, km 92.5, Zip code 18023-000, Sorocaba, SP, Brazil; E-Mail: vinazato@gmail.com (V.S.N.); 2 Universidade Federal do Pampa, UNIPAMPA, Av. Antônio Trilha, 1847, Zip code 97300-000, São Gabriel, RS, Brazil; E-Mail: charistonbelo@unipampa.edu.br (C.A.D.-B); 3 Universidade do Vale do Paraiba, UNIVAP, Av. Shishima Hifumi, 2911- Urbanova, Zip code 12244-000, São José dos Campos, SP, Brazil; E-Mail: jccogo@univap.br (J.C.C.); 4 Universidade Federal do Tocantins, UFT, Av. NS 15 ALC NO 14, 109 Norte, Zip code 77001-090, Porto Nacional, TO, Brazil; E-Mail: galdino@uft.edu.br (M.G.D.S.); 5 Universidade Estadual de Campinas, UNICAMP, R. Monteiro Lobato, 255, Zip code 13083-862, Campinas, SP, Brazil; E-Mail: hofling@unicamp.br (M.A.D.C.-H.)

**Keywords:** *Bothrops jararacussu*, *Crotalus durissus terrificus*, medicinal plant extract, myotoxicity, neurotoxicity

## Abstract

Extracts from *Dipteryx alata* bark obtained with different solvents (hexane, dichloromethane, ethyl acetate and methanol) were mixed *in vitro* with *Bothrops jararacussu* (Bjssu, 40 μg/mL) and *Crotalus durissus terrificus* (Cdt, 15 μg/mL) snake venoms, and applied to a mouse phrenic nerve-diaphragm preparation to evaluate the possible neutralization of venom effects. Cdt venom neurotoxic effect was not inhibited by any of the extracts, while the neurotoxic and myotoxic actions of Bjssu venom were decreased by the methanolic extract. This inhibition appears to be augmented by tannins. Dichloromethane bark extract inhibited ~40% of Bjssu venom effects and delayed blockade induced by Cdt. The methodology used to determine which extract was active allows inferring that: (i) phenolic acids and flavonoids contained in the methanolic extract plus tannins were responsible mostly for neutralization of Bjssu effects; (ii) terpenoids from the dichloromethane extract may participate in the anti-Cdt and anti-Bjssu venom effects; (iii) a given extract could not inhibit venoms from different species even if those belong to the same family, so it is improper to generalize a certain plant as antiophidian; (iv) different polarity extracts do not present the same inhibitory capability, thus demonstrating the need for characterizing both venom pharmacology and the phytochemistry of medicinal plant compounds.

## 1. Introduction

The use of medicinal plants for treating diseases, or to counteract envenoming effects caused by accidents with bees, wasps, scorpions, sea-anemones and snakes has long been a complementary and/or an alternative practice in substitution to conventional therapies [1]. In the case of snake envenomation, the mechanisms involved in the plants’ antiophidian effects are unclear; nevertheless it is quite certain that the targets of plant extracts are the venom components responsible for the severe effects of envenoming, such as phospholipases A_2,_ proteases [2], hemorrhagins [3,4], hyaluronidases [5], clotting serine-proteases [6], cytotoxins [7], myotoxins [8] and neurotoxins [9,10]. 

Plants constitute the largest reservoir of new drugs [11], and extraction, isolation and purification of their bioactive compounds are essential steps for prospection of new therapeutically active molecules [12]. There is a vast amount of literature reporting near 50 compounds isolated from medicinal plants which exhibit some degree of neutralization against the effects of snake venoms [13]. However, most medicinal plants lack phytochemical evaluation aimed at the isolation and identification of bioactive compounds against snake venom effects. 

In this regard, determination of specificity in the chemical interactions between the venom toxins and plant bioactive compounds is a key-point for such endeavors [14]. In this sense, of primordial importance is to implement measures for developing phytocomplexes exhibiting true pharmacological efficacy, and minor loss of therapeutic potential. For instance, rutin [15], tannin and coumarin [16], are plant-derived compounds known to be main-players in counteracting snake venom effect when present in the crude extract of the original plants’ leaves, but they significantly lose their antiophidian activity when isolated, as observed with the former [15]. Other essential steps include: (i) to have a perfect knowledge of clinical effects of victims of envenoming by a given snake species (e.g., pain, edema, inflammation, myonecrosis, hemorrhage, clotting disturbances, renal failure, neurotoxicity, *etc.*), and (ii) to have characterized the pharmacology of venom and/or their toxins (neurotoxins, metaloproteinases, myotoxins) [17,18]. 

An interesting point related to these issues is the belief that the efficacy of an antiophidian plant could be extended to venoms of all snake genera. Snake venoms are a complex mixture of components which can vary enormously among genera and even among species of the same genus. The purpose of the present study is to evaluate if bark extracts of *Dipteryx alata* Vogel obtained with different solvents (ranging from apolar to polar) have potential to counteract, *in vitro*, the toxic effects of snake venoms. The venoms of two different snake species from the Viperidae family were assayed: the South American rattlesnake* Crotalus durissus terrificus* venom, which preponderantly induces neurotoxic manifestations, and the *Bothrops jararacussu *venom, to which is accounted the myotoxic manifestations, besides pain, edema, disturbances in blood coagulation and acute renal failure [2,3,6,8]. The neurotoxicity (neurotransmission paralysis) and myotoxicity (muscle fibers necrosis) produced by these venoms in nerve-muscle preparation have been well described elsewhere [19,20] and the effect of *D. alata* to neutralize these effects will be evaluated. *Dipteryx alata* Vogel (Leguminosae-Mimosoideae, Fabaceae) has been considered a medicinal plant [21]; its fruits are comestible by animals [22] and humans [23]. The interest of this study relies on searching natural products from vegetal sources able to act as venom effects inhibitors and hence be used to improve serum therapy specially in terms of preventing local tissue damage. 

## 2. Results and Discussion

The search for bioactive molecules in plants used in folk medicine has been growing in the last few years. More than 40 compounds have been isolated from reported anti-snake venom plants and described in the literature [13]. These compounds are products of plant primary and secondary metabolism which are believed to have similar pharmacological potential as the ones found in animal organisms. They can likely be peptides and work as stimulants of the immunological system [24]; be phenolic compounds able to complex with protein by hydrogen bonding and generically function as antioxidant agents [25]; be steroids and terpenoids and act as endocrine function regulators or as signaling molecules by forming complexes with proteins through van der Waals and/or hydrophobic forces [26]. 

In this study, thin layer chromatography (TLC) was applied for obtaining a chromatographic profile of hydroalcoholic extract (HA) from *D. alata* barks, since pilot assays have shown a neutralizing ability of HA from *D. alata* barks against the neuromuscular blockade induced by *Bothrops jararacussu* snake (Bjssu) venom. TLC is useful to detect such compound by fingerprinting because it allows for separating components and visually comparing them with standard phytochemicals [25]. 

**Figure 1:**
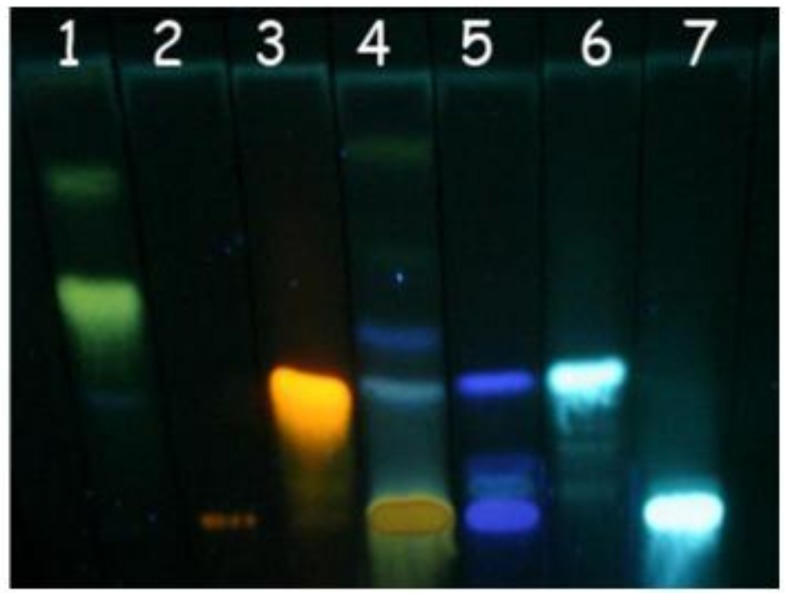
Thin Layer Chromatography. *D. alata* hydroalcoholic extract and phytochemical standards. Solvent system - acetone: chloroform: formic acid (10:75:8 v/v). Developer: NP/PEG. 1. Apigenin, 2. Rutin, 3. Quercetin, 4. *D. alata* hydroalcoholic extract, 5. Tannic acid, 6. Caffeic acid, and 7. Chlorogenic acid. The chromatographic profile exhibits yellow –orange (flavonoids) and blue colored (phenolic acids) spots characteristic of phenolic compounds.

Figure 1 shows the chromatogram obtained with an acetone-chloroform-formic acid (10:75:8, v/v) eluent system. The retention factor (Rf) of extracts was compared to the Rf of standards in order to determine the type of phytochemical present. The Rfs obtained in this circumstances were: 0.34, 0.57 and 0.82 for apigenin (**1**), 0 for rutin (**2**), 0.31 for quercetin (**3**), 0.05 and 0.34 for tannic acid (**5**), 0.38 for caffeic acid (**6**) and 0 for chlorogenic acid (**7**). Note that HA (4) extract from *D. alata* showed spots with Rf similar to caffeic and tannic acids, indicating the presence of phenolic acids, typically represented by blue colors. The spot at the place of application exhibited a yellow color, indicating the presence of flavonoids. 

**Figure 2:**
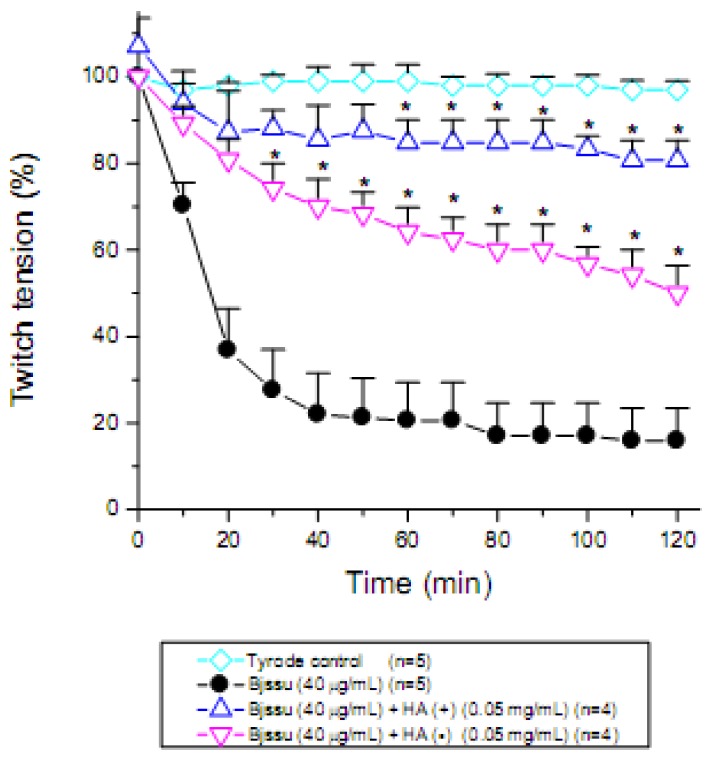
Pharmacological assays to determine the effect of tannin in the hydroalcoholic (HA) extract of *D. alata* barks on mouse phrenic nerve-diaphragm preparations stimulated indirectly. Note that both the extract containing tannin [HA(+)] and the extract free of tannin [HA(-)] significantly improved the muscle twitch tension and hence decreased neuromuscular blockade promoted by *Bothrops jararacussu* (Bjssu) venom alone. However, HA(+) showed more efficicacy than HA (-). Each point represents the mean ± S.E.M. of the number of experiments (n) showed in the legend. *p < 0.05 compared to venom.

As the TLC plate provided suggestive evidence of the presence of phenolic acids in the hydroalcoholic extract (HA), the role of tannins, quantified at 0.79058 mg/mL (~80%), was biomonitored through its presence [HA(+)] or absence [HA(-)] in the extract. Figure 2 shows these two conditions. When tannins are present [HA(+)] the extract is able to neutralize the neuromuscular blockade promoted by Bjssu venom (n = 4). The protective effect of commercially available tannic acid was already demonstrated *in vivo *after subcutaneous injection in mice envenomed with *Crotalus adamanteus* venom (decreased hemorrhage, creatine kinase release and lethal activity) [27], or after its pre-incubation for 1 h at 37 ºC with *Naja kaouthia* Lesson (Elapidae) venom before intravenous injection in mice [28]. 

In HA(-)tannin free extract there was a time-dependent gradual decrease in the protection level against Bjssu venom (n = 4) when compared to the promoted by HA plus tannins [HA(+) extract]. At the end of the incubation time (120 min) there was approximately a 50% decline in the protection of the extract free of tannins [HA(-) extract]. However, in comparison with venom effect alone, the HA(-) tannin free extract still maintained about 25% protection (from 40 min to 120 min incubation time), indicating that components in the HA extract other than tannins participate in the protection, although not as effectively as when tannin is present. Figure 2 shows that there was a significant difference between the HA(-) tannin free extract and HA(+) tannin extract within the 40–120 min time interval of incubation in their ability to counteract venom effects. We suggest that the protective activity of antiophidian tannin-rich plants can be likely attributed to tannin physico-chemical properties capable of promoting protein precipitation. In regard to this issue, tannins and tannic acid share identical properties since both promote the precipitation of proteins [29]. Tannic acid has been already proved to precipitate proteins present in several snake venoms including venoms of the Elapidae family (*Naja kaouthia*) [28] and the Viperidae family (*C.d. terrrificus, Bothrops jararacussu*) [16] and *Crotalus adamanteus* [27]. 

**Figure 3:**
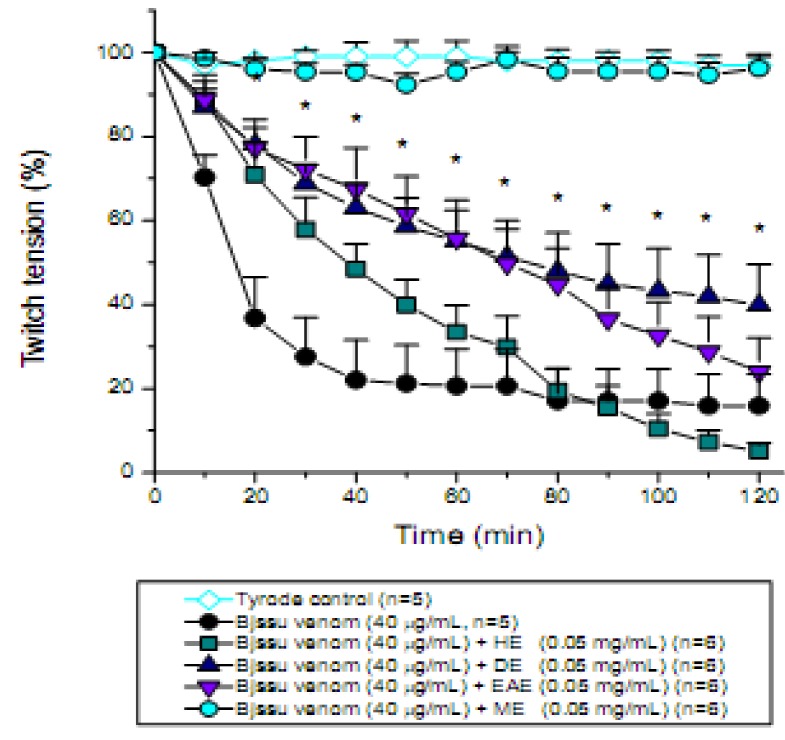
Pharmacological assays on mouse phrenic nerve-diaphragm preparations to determine the twitch response under electric indirect stimuli after incubation the preparation with 40 µg/mL *B. jararacussu* (Bjssu) venom pre-incubated for 30 min with the *D. alata* barks extract (0.05 mg/mL) using different solvents (HE, hexane; DE, dichloromethane; EAE, ethyl acetate; ME, methanol). Note that methanolic extract 100% prevented the neuromuscular blockade promoted by Bjssu venom alone whereas dichloromethane extract significantly decreased by ~15% the neuromuscular paralysis. See also that the hexane extract accentuated the toxic effect of venom. Each point represents the mean ± S.E.M. of the number of experiments (n) showed in the legend. *p < 0.05 compared to venom.

**Figure 4:**
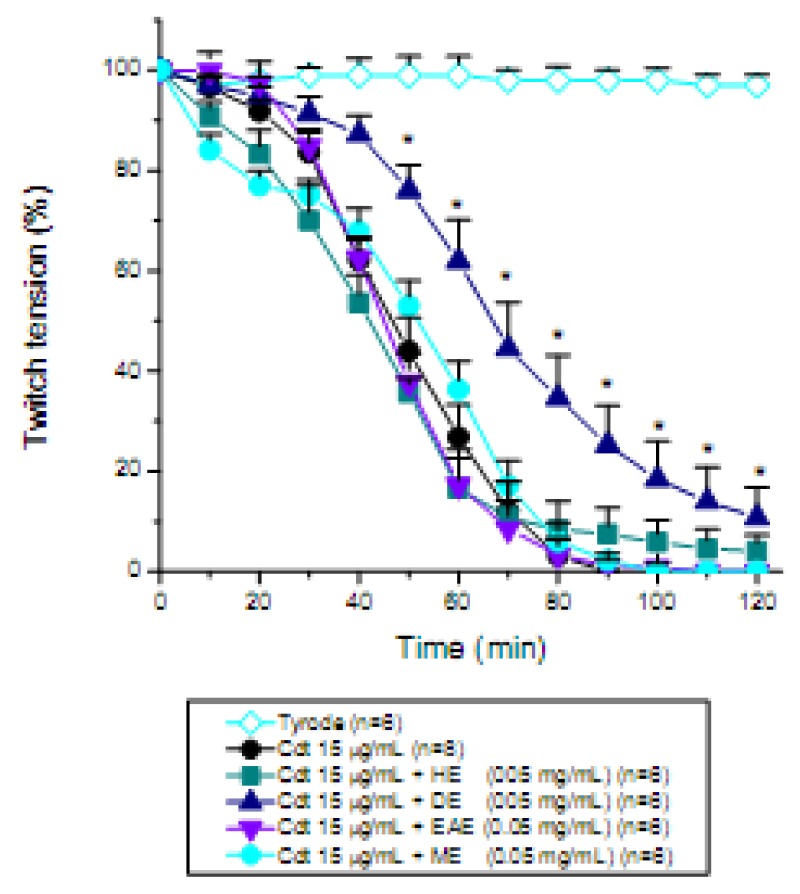
Pharmacological assays on mouse phrenic nerve-diaphragm preparations to determine the twitch response under electric indirect stimuli after incubation the preparation with 15 µg/mL *Crotalus durissus terrificus* (Cdt) venom pre-incubated for 30 min with the *D. alata* barks extract (0.05 mg/mL) using different solvents (HE, hexane; DE, dichloromethane; EAE, ethyl acetate; ME, methanol). Note that none of the extracts were able to prevent the total neuromuscular blockade promoted by Cdt venom, except DE which did not prevent the blockade but instead delayed temporarily its appearance. Each point represents the mean ± S.E.M. of the number of experiments (n) showed in the legend. *p < 0.05 compared to venom.

The continuous extraction of organic constituents from dried plant barks was carried out with a Soxhlet apparatus by using a range of solvents (from apolar to polar). Such different extracts were used in the neutralization assays to identify the extract with best antiophidian efficacy. The findings showed that the different solvent extracts (hexane, HE; dichloromethane, DE; ethyl acetate, EAE; or methanol, ME) exhibited differential biological activities, when tested pharmacologically against the venoms of two snakes,* B. jararacussu* (Figure 3) and *C. d. terrificus* (Figure 4), in relation to twitch tension recording of the mouse phrenic nerve-diaphragm (PND) preparation. For instance, hexane removes apolar constituents such as terpenes and chlorophylls, whereas dichloromethane is a solvent used to isolate chlorophylls and components with low polarities. On the other side, ethyl acetate is a solvent which separates low chlorophyll containing-components, whereas methanol is a solvent which separates components with high polarity such as flavonoids and phenolic acids [25].

Figure 3 shows the pharmacological profile of the *in vitro* neutralization assays after pre-incubation for 30 min of each 0.05 mg/mL (n = 6) HE, DE, EAE, and ME extract with Bjssu venom (40 µg/mL) prior to muscle twitch tension measurements. Note a partial protection promoted by dichloromethane extract (DE, *p < 0.05 compared to venom). However, the most notable protection was promoted by the methanolic extract (ME, p < 0.05, as can be seen by absence of difference in relation to Tyrode control preparation). 

When each extract (HE, DE, EAE or ME) from *D. alata* was pre-incubated with *C. d. terrificus* (Cdt) venom (15 µg/mL) and further applied to a mouse phrenic nerve-diaphragm preparation, practically any protection was noticed (Figure 4). The better performance for counteracting Cdt venom neuromuscular blocking effects was obtained with DE extract (*p < 0.05, from 50 min to 120 min). Clearly, one of the venoms was not inhibited (Cdt), while the other (Bjssu) decreased its toxic potential when preincubated with the whole methanolic extract. This inhibition strongly appears to be mediated mostly by tannins, and by phenolic acids and flavonoids. 

Currently, the antiophidian effect of a purified compound isolated from a plant extract can present a diminished efficiency compared to the whole extract. Recently, we showed that rutin was* an important component of the methanolic extract of Casearia** sylvestris** Sw. and possibly the main responsible for the neutralization effects exhibited by plant leaves extract against the *in vitro* neuromuscular actions of both the Bjssu venom and its major toxin, the basic phospholipase A_2 _bothropstoxin-I *[15].* However, rutin by itself when tested in nerve-muscle preparation was unable to protect against both venom and toxin, suggesting that likely the pharmacological protection was effective only when phytochemical-venom complexes are preserved in the extract*. **

In agreement, Melo *et al*. [16] demonstrated the importance of formation of such complexes by comparing the action of two commercial isolated phytochemical standards (tannic acid and coumarin) and two extracts of plants rich in these compounds effective against Bjssu and Cdt venoms. The authors observed that during tannic acid pre-incubation with venoms a precipitate, resulting from tannin+venom protein complexation was formed, which was more intense with Cdt than with Bjssu venom. Such tannin-Cdt and -Bjssu venom protein complexes were proved to be a fundamental step in the loss of venom toxicity. These intriguing results led the authors to apply the same protocol using selected hydroalcoholic extracts from *Plathymenia reticulata*, which has tannins and flavonoids [30] and from *Mikania laevigata*, which has coumarin [31]. Both hydroalcoholic extracts were able to inhibit both Bjssu and Cdt venom toxicity. When tannin traces were removed from both hydroalcoholic extracts only the *M. laevigata*, but not *P. reticulata* one, attenuated the effects of Bjssu venom, but not of Cdt venom. From the isolated compounds, only tannic acid alone was able to neutralize the venoms’ neuromuscular effects. Interestingly, coumarin did not form a precipitate when added to either venom and did not neutralize their effects. The authors then concluded that the efficiency of a plant isolated compound to inactivate snake venom action may depend on the mechanism of action of the selected venom. However, based on the results obtained with coumarin, the authors suggest that the plant also plays a key role in such a mechanism. 

**Figure 5:**
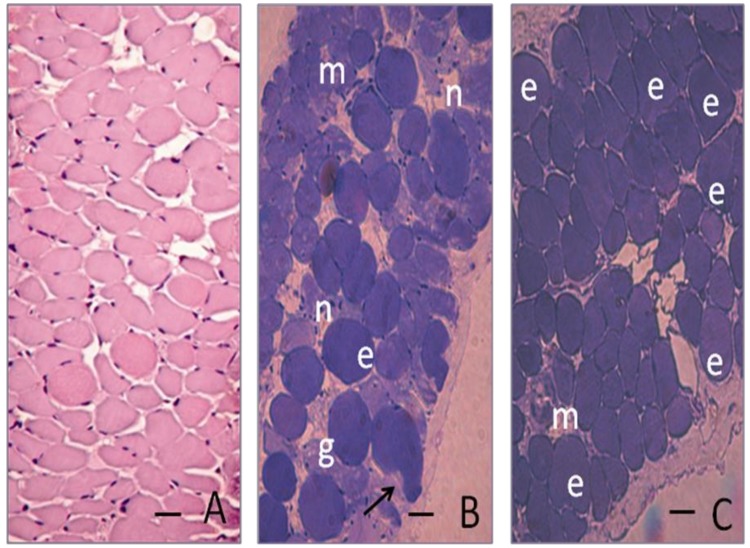
Cross-sections (2 μm thick) of diaphragm after a 120 min incubation and indirect stimulation though the phrenic nerve. (A) Control-sham diaphragm preparation of paraffin embedded sections stained with Hematoxylin-Eosin. (B and C) Preparations embedded in historesin and stained with Toluidine blue. (B) Muscle incubated with 40 μg/mL Bjssu venom shows 56 ± 6% of fibers exhibiting various myonecrotic (**m**) states characterized by edema (**e**), delta lesion (**arrow**), presence of vacuoles, atrophy of the muscle fibers, sarcolemmal disruption with nuclei (**n**) dispersion, and “ghost” cells (**g**) visualized by spaces optically empty. Area with extensive myonecrosis assumed a hyaline aspect. (C) Muscle incubated with 40 μg/mL *Bothrops jararacussu* (Bjssu) venom plus methanolic extract shows fibers maintaining its characteristic polygonal profile, despite a number of them were edematous (**e**). A slow percentage of them (17 ± 2%) showed myonecrosis (**m**). Bar = 50 μm.

The failure of *D. alata* extracts in protecting against the neuromuscular blockade caused by Cdt venom led us to analyze histologically only the diaphragm incubated with Bjssu venom and Bjssu venom plus extracts. In nerve-muscle preparations exposed to either Tyrode or ME extract, the muscle fibers were well-preserved, showing slight changes not exceeding 10 ± 2% of damaged fibers. Moreover, the changes were mainly related to loss of the typical cell cross-sectional polygonal profile (Figure 5A), and totally different from the characteristic lesions seen with venom (atrophy of the muscle fibers, hypercontraction, sarcolemma disruption and condensation and/or lysis of the myofibrils). Figure 5B shows cross-sections of muscle fibers of PND exposed for 120 min under indirect stimulation to Bjssu venom (40 µg/mL, 56 ± 6% of damaged fibers, n = 4) or after venom *in vitro* pre-incubation with methanolic extract (C, Bjssu + ME, 17 ± 2% of damaged fibers, p < 0.05, n = 4). 

Note in panel B (Bjssu-treated preparation) the presence of representative myonecrotic cells (m), fibers heterogeneous in size, some of them with edematous aspect (e) and which lost its polygonal cross profile, or presented delta lesions (arrow), or vacuoles indicating myofibril condensation. Also, sarcolemma disruption and loss of intracellular material indicates presence of areas of pulverulent sarcoplasm or “ghost” cells (g), and displaced nuclei (n), all these characteristics indicative of severe myonecrosis. Note in panel C (venom + ME), that such myonecrotic (m) characteristics decreased notably, the majority of cells maintained their polygonal profile and only a few were edematous (e), indicating that the methanolic extract prevented just the following step, the membrane disruption.

The literature clearly shows the effectiveness of tannic acid in decreasing the venom toxicity of some Viperidae species such as *C. d. terrificus* [16], *C. adamanteus *[27] and* B. jararacussu *[16], and Elapidae, *i.e., N. kaouthia* [28] snake venoms, likely by precipitating venom proteins [16] and hence promoting its denaturation and loss of toxicity. Like tannic acid, tannins from three different plant species demonstrated to be a crucial antiophidian compound present in ME extract, and in HA extract [16]. Its removal from the extract reduced significantly the protective anti-toxic effect (protein denaturation) against Bjssu venom. Tannins, in contrast to tannic acid, may have different molecular configurations and consequently have the ability to bind with different compounds. Like tannic acid, tannins are also able to precipitate proteins [29]. Since tannins are heterogeneous phytocomplexes, the possibility that they exhibit different selectivity in relation to precipitation properties of proteins from Cdt and Bjssu venom cannot be discounted. 

In this regard, as aforementioned, it is mandatory to know the pharmacology of the venoms tested. In fact, there are differences in clinical signs, local and systemic effects induced by *C. d. terrificus* and *B. jararacussu* snakebite accidents; these differences have been attributed to differences in composition and mechanism of action of the venoms of the two Viperidae species [18]. *C. d. terrificus* venom is highly neurotoxic, its neurotoxicity being attributed to crotoxin, a neurotoxin with PLA_2_ activity [32,33,34], whereas *Bothrops jararacussu* venom is preponderantly myotoxic, being this attributed to its major myotoxin bothropstoxin-I [35]. Crotoxin and bothropstoxin-I are phospholipases; the former is an Asp49PLA_2_, catalytically active whereas the latter is a Lys49PLA_2_, catalytically inactive. In the present study, dichloromethane extract was able to delay (but not prevent) the neuromuscular blockade induced by Cdt venom. Probably, terpenoids contained in this extract are responsible for such effects. Cavalcante *et al*. [36] showed the ability of aqueous leaves extract of *Casearia sylvestris* to counteract the actions of PLA_2_ present in snake venoms, such as crotoxin and bothropstoxin-I, an ability that *D. alata* bark extracts seem not to have, or they are simply incapable of neutralizing Asp49PLA_2_. 

Thus, by using a nerve-muscle preparation we showed that the antiophidian principle contained in the barks extract of *D. alata* can be active, depending on the solvent used. It was also demonstrated that the efficiency of a given extract against a particular venom could not be extrapolated for all snake venoms. Here, the methanolic extract of *D. alata* barks counteracted the *in vitro* neuromuscular paralysis and myonecrosis produced by *B. jararacussu *venom, but failed in relation to neuromuscular paralysis produced by *C. d. terrificus*. We also showed that tannins contained in the *D. alata* barks extract have a prominent role in the neutralization of *B. jararacussu* venom effects.

## 3. Experimental

### 3.1. Animals

Male Swiss white mice (26–32 g) were supplied by Anilab (Animais de Laboratório, Paulínia, S.P., Brazil). The animals were housed at 25 ± 3 ºC on a 12 h light/dark cycle and had access to food and water *ad libitum*. This project (protocol n^o^ A012/CEP/2006) was approved by the institutional Committee for Ethics in Research of Vale do Paraiba University (UNIVAP), and the experiments were carried out according to the guidelines established by the Brazilian College for Animal Experimentation (COBEA).

### 3.2. Venoms

*Bothrops jararacussu* (Bjssu) and *Crotalus durissus terrificus* (Cdt) snakes were captured in the Southeast of Brazil, state of São Paulo and transferred to the “Serpentário do Centro de Estudos da Natureza” of the Vale do Paraíba University - UNIVAP (São José dos Campos, SP, Brazil). The venoms were obtained from adult specimens living in captivity for more than year. 

### 3.3. Dipteryx alata Vogel extracts 

The barks of *D. alata* were collected in August 2007 in Porto Nacional city, Tocantins State, Brazil. The taxonomist who identified the plant was Dr. Solange F. Lolis from the Herbarium of the Universidade Federal do Tocantins, Palmas, To, Brazil. A specimen was deposited (protocol IAC 50629) at the Herbarium of Instituto Agronômico de Campinas, Campinas, SP, Brazil. Soxhlet vessels containing 55 g of bark powder (after drying by using a Marconi forced air circulation apparatus, and grinding to 10 mesh using a Wiley type Marconi, MA 340 model macromill) were filled successively with 300 mL of different solvents ranging, from apolar to polar, as follows: hexane, dichloromethane, ethyl acetate (all from Synth^®^, Brazil) and methanol (Ecibra^®^, Brazil). Then, the solvents were evaporated to dryness and the dried extract powders (0.6237, 0.4251, 0.7661, and 2.6523 g corresponding to hexane, dichloromethane, ethyl acetate and methanol, respectively) stored at room temperature and protected from light and humidity until the assays were performed. Part of dried barks was macerated (200 g, during 5 days) in 2 L of 70% ethanol and the suspension was then percolated (under protection against light) at 20 drops/min, resulting in a 20% (m/v) hydroalcoholic extract (HA) [37]. 

### 3.4. Protein precipitation assay and tannins determination 

Hydroalcoholic extract (HA) above obtained was used for protein precipitation assays. The proteins in the extract solution were precipitated according to Hagerman and Butler [37], dissolved in sodium dodecyl sulfate (Sigma)/triethanolamine (Merck), complexed in FeCl_3_ and read at 510 nm for tannin determination [38,39]. The tannin concentration in the sample was measured through a standard curve obtained by a polynomial regression y = 1.754 x – 0.1253 (R^2 ^= 0.9971). Tannic acid was used for the standard curve. All the solutions were analyzed in triplicate. The supernatant of tannins free extract was used in the venom neutralizing assays. This assay was done to verify whether the protection provided by the bark extracts obtained by the different solvents was due to the extract itself or was due to the presence of tannin in the barks of *D. alata.*

### 3.5. Thin layer chromatography 

Aliquots of *D. alata* hydroalcoholic (HA) extracts were spotted on thin-layer silica gel plates (0.3 mm thick, Merck, Germany) and compared with a collection of reference phytochemicals [25]. The solvent system consisted of acetone-chloroform-formic acid, 10:75:8, v/v. The phytochemical groups used as standards (suspended in 1% methanol m/v, P.A. solution, Sigma Chemical Co., St. Louis, MO, USA) were flavonoids (apigenin, quercetin, and rutin) and phenolic acids (tannic, caffeic and chlorogenic acids). The separated spots were visualized (under UV light at 360 nm) with NP/PEG as follows: 5% (v/v) ethanolic NP (diphenylboric acid 2-aminoethyl ester, Sigma^®^ Chemical Co., St. Louis, MO, USA) followed by 5% (v/v) ethanolic PEG 4000 (polyethylene glycol 4000, Synth^®^ Chemical Co., São Paulo, SP, Brazil). For determination of type of phytochemical contained in each extract, the retention factor (Rf) of extract spots were compared with the Rf of each standard.

### 3.6. Mouse phrenic nerve-diaphragm muscle (PND) preparation

The phrenic nerve-diaphragm muscle [40] was obtained from mice previously anesthetized with halotane and sacrificed by exsanguination. The diaphragm was removed and mounted under a tension of 5 g in a 5 mL organ bath containing Tyrode solution with the following composition (mM): NaCl 137, KCl 2.7, CaCl_2_ 1.8, MgCl_2_ 0.49, NaH_2_PO_4_ 0.42, NaHCO_3_ 11.9 and glucose 11.1. After equilibration with the carbogen aeration mixture of 95% O_2_/5% CO_2_, the pH of this solution was set at 7.0. The preparations were stimulated indirectly with supramaximal stimuli (4 × threshold, 0.1 Hz, 0.2 ms) delivered from an ESF-15D (Ribeirão Preto, Brazil) stimulator to the nerve through bipolar electrodes. Isometric twitch tension was recorded with a force displacement transducer (cat. 7003, Ugo Basile) coupled to a physiograph 2-Channel Recorder Gemini (cat. 7070, Ugo Basile) via a Basic Preamplifier (cat. 7080, Ugo Basile). PND preparations were allowed to stabilize for at least 20 min before addition of one of the following solutions: Tyrode solution (control, n = 5); 40 µg/mL Bjssu (n = 5) or 15 µg/mL Cdt (n = 8) venoms, in concentrations previously chosen. The neutralization assays were done by mixing together the venom plus 0.05 mg/mL of each of the extracts (hexane, HE; dichloromethane, DE; ethyl-acetate, EAE or methanol, ME; n = 6 each) 30 min before addition to the neuromuscular preparation. The extract concentration was chosen after a dose-response curve with 0.05, 0.1 and 0.2 mg/mL of *D. alata* hydroalcoholic extract (not shown), where the first one showed minor effect on the basal response of preparation. 

### 3.7. Histological and quantitative study

At the end of experiments, preparations exposed to Tyrode (controls), Bjssu venom alone or to mixture of Bjssu venom plus ME, (during 120 min, n = 4 each) were fixed in Bouin’s fixative and routinely processed for embedding in paraffin or Historesin (Leica Instruments Gmbh, Nubloch/Heidelberg). At least 2 cross-sections, 2 µm thick, of diaphragm muscle were stained with 0.5% toluidine blue for examination by light microscopy. Morphological damage was quantified in Tyrode- (control, paraffin sections stained with Hematoxylin-Eosin) and venom-incubated preparations (n = 4) (historesin sections stained with Toluidine blue) by counting the number of fibers with lesions (edema, intense myonecrosis characterized by atrophy of the muscle fibers, hyaline aspect, sarcolemmal disruption and lysis of the myofibrils) and this value was expressed as percentage of the total number of damaged muscle cells divided by the total number of cells in three non-overlapping, non-adjacent areas of each section [20]. At the end 60 areas were examined resulting in a total counting of 584 fibers. 

### 3.8. Statistical analysis

The results were expressed as the mean ± S.E.M. Student’s *t*-test or the ANOVA and Tukey (*post hoc*) tests were used for data comparison. The significance level was set at 5%.

## 4. Conclusions 

The phenolic acids and flavonoids contained in the methanolic extract plus tannins of *D. alata* extracts were the main players in the neutralization of Bjssu venom effects, whereas the terpenoids (from dichloromethane extract) displayed partial activity against the effects of Cdt and Bjssu venom. Excluding tannins, which were proven to act by precipitating proteins contained in the venom and hence destroying its toxicity, other compounds might be acting by forming a phytocomplex (in this case more than one substance has a protective role), as inferred by the partial protection (25%) seen in the extract free of tannins. Independent on the mechanism(s) we suggest that methanolic extract of *D. alata* barks is a promising clinical approach to be used as a topical first aid treatment at the bite site in *B. jararacussu* envenoming. Experimental *in vivo* studies are necessary to evaluate deeply its ability to prevent the fast developing local myonecrosis. 

## References

[1] Mirshafiey A. (2007). Venom therapy in multiple sclerosis. Neuropharmacology.

[2] Borges M.H., Soares A.M., Rodrigues V.M., Oliveira F., Fransheschi A.M., Rucavado A., Giglio J.R., Homsi-Brandeburgo M.I. (2001). Neutralization of proteases from *Bothrops* snake venoms by the aqueous extract from *Casearia sylvestris* (Flacourtiaceae). Toxicon.

[3] Da Silva J.O., Coppede J.S., Fernandes V.C., Sant’Ana C.D., Ticli F.K., Mazzi M.V., Giglio J.R., Pereira O.S., Soares A.M., Sampaio S.V. (2005). Antihemorrhagic, antinucleolytic and other antiophidian properties of the aqueous extract from *Pentaclethra macroloba*. J. Ethnopharmacol..

[4] Nishijima C.M., Rodrigues C.M., Silva M.A., Lopes-Ferreira M., Vilegas W., Hiruma-Lima C.A. (2009). Anti-hemorrhagic activity of four Brazilian vegetable species against *Bothrops jararaca* venom. Molecules.

[5] Girish K.S., Kemparaju K. (2005). Inhibition of *Naja naja* venom hyaluronidase by plant-derived bioactive components and polysaccharides. Biochemistry.

[6] Oliveira C.Z., Maiorano V.A., Marcussi S., Sant’Ana C.D., Januário A.H., Lourenço M.V., Sampaio S.V., França S.C., Pereira O.S., Soares A.M. (2005). Anticoagulant and antifibrinogenolytic properties of the aqueous extract from *Bauhinia forficata* against snake venoms. J. Ethnopharmacol..

[7] Aguiyi J.C., Guerranti R., Pagani R., Marinello E. (2001). Blood chemistry of rats pretreated with *Mucuna pruriens* seed aqueous extract MP101UJ after *Echis carinatus* venom challenge. Phytother. Res..

[8] Mors W.B., do Nascimento M.C., Parente J.P., da Silva M.H., Melo P.A., Suarez-Kurtz G. (1989). Neutralization of lethal and myotoxic activities of South American rattlesnake venom by extracts and constituents of the plant *Eclipta prostrata* (Asteraceae). Toxicon.

[9] Ratanabanangkoon K., Cherdchu C., Chudapongse P. (1993). Studies on the cobra neurotoxin inhibiting activity in an extract of *Curcuma* sp. (Zingiberaceae) rhizome. Southeast Asian J. Trop. Med. Public Health.

[10] Maiorano V.A., Marcussi S., Daher M.A.F., Oliveira C.Z., Couto L.B., Gomes A.O., França S.C., Soares A.M., Pereira O.S. (2005). Antiophidian properties of the aqueous extract of *Mikania glomerata*. J. Ethnopharmacol..

[11] Sévenet T. (1991). Looking for new drugs: what criteria?. J. Ethnopharmacol..

[12] Bezerra J.A., Campos A.C., Vasconcelos P.R., Nicareta J.R., Ribeiro E.R., Sebastião A.P., Urdiales A.I., Moreira M., Borges A.M. (2006). Extract of *Passiflora edulis* in the healing of colonic anastomosis in rats: tensiometric and morphologic study. Acta Cir. Bras..

[13] Soares A.M., Ticli F.K., Marcussi S., Lourenço M.V., Januário A.H., Sampaio S.V., Giglio J.R., Lomonte B., Pereira P.S. (2005). Medicinal plants with inhibitory properties against snake venoms. Curr. Med. Chem..

[14] Mahmood A., Ahmad M., Jabeen A., Zafar M., Nadeem S. (2005). Pharmacognostic studies of some indigenous medicinal plants of Pakistan. Ethnobotanical Leaflets.

[15] Cintra-Francischinelli M., Silva M.G., Andréo-Filho N., Gerenutti M., Cintra A.C.O., Giglio J.R., Leite G.B., Cruz-Höfling M.A., Rodrigues-Simioni L., Oshima-Franco Y. (2008). Antibothropic action of *Casearia sylvestris* Sw (Flacourtiaceae) extracts. Phytother. Res..

[16] Melo R.S., Farrapo N.M., Rocha-Junior D.S., Silva M.G., Cogo J.C., Dal Belo C.A., Rodrigues-Simioni L., Groppo F.C., Oshima-Franco Y., Keller R.B. (2009). Antiophidian mechanisms of medicinal plants. Flavonoids: Biosynthesis, Biological Effects and Dietary Sources.

[17] Cintra-Francischinelli M., Pizzo P., Rodrigues-Simioni L., Ponce-Soto L.A., Rossetto O., Lomonte B., Gutiérrez J.M., Pozzan T., Montecucco C. (2009). Calcium imaging of muscle cells treated with snake myotoxins reveals toxin synergism and presence of acceptors. Cell Mol. Life Sci..

[18] Ministério da Saúde do Brasil (2001). Manual de Diagnóstico e Tratamento de Acidentes Por Animais Peçonhentos.

[19] Oshima-Franco Y., Hyslop S., Prado-Franceschi J., Cruz-Höfling M.A., Rodrigues-Simioni L. (1999). Neutralizing capacity of antisera raised in horses and rabbits against *Crotalus durissus terrificus* (South American rattlesnake) venom and its main toxin, crotoxin. Toxicon.

[20] Oshima-Franco Y., Leite G.B., Silva G.H., Cardoso D.F., Hyslop S., Giglio J.R., da Cruz-Höfling M.A., Rodrigues-Simioni L. (2001). Neutralization of the pharmacological effects of bothropstoxin-I from *Bothrops jararacussu* (jararacuçu) venom by crotoxin antiserum and heparin. Toxicon.

[21] Dos Santos M.G., Lolis S.F., Dal Belo C.A. (2006). Ethnobotanic Survey of Two Remaining Communities of Black-Africans of Jalapão Region Tocantins State.

[22] Lorenzi H. (1992). Árvores Brasileiras: Manual de Identificação e Cultivo de Plantas Arbóreas Nativas do Brasil.

[23] Togashi M., Sgarbieri V.C. (1995). Avaliação nutricional da proteína e do óleo de semente de baru (*Dipteryx alata* Vog.). Ciênc. Technol. Aliment..

[24] Guerranti R., Aguiyi J.C., Néri S., Leoncini R., Pagani R., Marinello E. (2002). Proteins from *Mucuna pruriens* and enzymes from *Echis carinatus* venom: characterization and cross-reactions. J. Biol. Chem..

[25] Harborne J.B. (1998). Phytochemical Methods: A Guide to Modern Techniques of Plants Analysis.

[26] Mors W.B., do Nascimento M.C., Parente J.P., da Silva M.H., Melo P.A., Suarez-Kurtz G. (2000). Plant natural products active against sanke bite – the molecular approach. Phytochemistry.

[27] Kuppusamy U.R., Das N.P. (1993). Protective effects of tannic acid and related natural compounds on *Crotalus adamanteus* subcutaneous poisoning in mice. Pharmacol. Toxicol..

[28] Pithayanukul P., Ruenraroengsak P., Bavovada R., Pakmanee N., Suttisri R. (2007). *In vitro* investigation of the protective effects of tannic acid against the activities of *Naja kaouthia* venom. Pharm. Biol..

[29] Haslam E. (2007). Vegetable tannins-lessons of a phytochemical lifetime. Phytochemistry.

[30] Fernandes T.T., dos Santos A.T., Pimenta F.C. (2005). Atividade antimicrobiana das plantas. Rev. Patol. Trop..

[31] Dos Santos S.C., Krueger C.L., Steil A.A., Kreuger M.R., Biavatti M.W., Wisniewski A. (2006). LC characterisation of guaco medicinal extracts, *Mikania laevigata* and *M. glomerata*, and their effects on allergic pneumonits. Planta Med..

[32] Bon C., Kini R.M. (1997). Multicomponent neurotoxic phospholipases A_2_. Venom Phospholipase A_2_ Enzymes: Structure, Function and Mechanism.

[33] Chang C.C., Lee J.D. (1977). Crotoxin, the neurotoxin of South American rattlesnake venom, is a presynaptic toxin acting like beta-bungarotoxin. Naunyn Schmiedebergs Arch. Pharmacol..

[34] Slotta K.H., Fraenkel-Conrat H. (1938). Schlangengiffe, III: Mitteilung Reiningung und crystallization des klappershclangengiffes. Ber. Dtsch. Chem. Ges..

[35] Homsi-Brandeburgo M.I., Queiroz L.S., Santo-Neto H., Rodrigues-Simioni L., Giglio J.R. (1988). Fractionation of *Bothrops jararacussu* snake venom: partial chemical characterization and biological activity of bothropstoxin. Toxicon.

[36] Cavalcante W.L.G., Campos T.O., Dal Pai-Silva M.D., Pereira O.S., Oliveira C.Z., Soares A.M., Gallaci M. (2008). Neutralization of snake venom phospholipase A_2_ toxins by aqueous extract of *Casearia sylvestris* (Flacourtiaceae) in mouse neuromuscular preparation. J. Ethnopharmacol..

[37] Portuguese Pharmacopoeia Committee (2002). Portuguese Pharmacopoeia.

[38] Hagerman A.E., Butler L.G. (1978). Protein precipitation method for the quantitative determination of tannins. J. Agric. Food Chem..

[39] Hagerman A.E., Butler L.G. (1989). Choosing appropriate methods and standards for assaying tannins. J. Chem. Ecol..

[40] Bülbring E. (1946). Observation on the isolated phrenic nerve diaphragm preparation of the rat. Br. J. Pharmacol..

